# 

**Published:** 1881-04

**Authors:** 


					﻿APRIL, 1881.
TWENTY-EIGHTH YEAR.
HAI.LS

OF

E. H. GIBBS, A.M., M.D., Editor,
No. 141 EIGHTH STREET (Near Broadway), NEW YORK.
TERMS: $2 a Year, or $1 for Six Months. Single nnmher, 20 ctsJ
Entered as Second class Matter In the Post-Office In New York.
				

